# Hearing handicap in patients with chronic kidney disease: a study of the different classifications of the degree of hearing loss^☆^

**DOI:** 10.1016/j.bjorl.2016.08.008

**Published:** 2016-09-10

**Authors:** Klinger Vagner Teixeira da Costa, Sonia Maria Soares Ferreira, Pedro de Lemos Menezes

**Affiliations:** a Hospital Vida, Centro Universitário CESMAC, Programa de Pós-graduação, Maceió, AL, Brazil; b Universidade Estadual de Ciências da Saúde de Alagoas, Maceió, AL, Brazil

**Keywords:** Chronic kidney disease, Hearing loss, Audiometry, Doença renal crônica, Perda auditiva, Audiometria

## Abstract

**Introduction:**

The association between hearing loss and chronic kidney disease and hemodialysis has been well documented. However, the classification used for the degree of loss may underestimate the actual diagnosis due to specific characteristics related to the most affected auditory frequencies. Furthermore, correlations of hearing loss and hemodialysis time with hearing handicap remain unknown in this population.

**Objective:**

To compare the results of Lloyd's and Kaplan's and The Bureau Internacional d’Audiophonologie classifications in chronic kidney disease patients, and to correlate the averages calculated by their formulas with hemodialysis time and the hearing handicap.

**Methods:**

This is an analytical, observational and cross-sectional study with 80 patients on hemodialysis. Tympanometry, speech audiometry, pure tone audiometry and interview of patients with hearing loss through Hearing Handicap Inventory for Adults. Cases were classified according to the degree of loss. The correlations of tone averages with hemodialysis time and the total scores of Hearing Handicap Inventory for Adults and its domains were verified.

**Results:**

86 ears (53.75%) had hearing loss in at least one of the tonal averages in 48 patients who responded to Hearing Handicap Inventory for Adults. The Bureau Internacional d’Audiophonologie classification identified a greater number of cases (*n* = 52) with some degree of disability compared to Lloyd and Kaplan (*n* = 16). In the group with hemodialysis time of at least 2 years, there was weak but statistically significant correlation of The Bureau Internacional d’Audiophonologie classification average with hemodialysis time (*r* = 0.363). There were moderate correlations of average The Bureau Internacional d’Audiophonologie classification (*r* = 0.510) and tritone 2 (*r* = 0.470) with the total scores of Hearing Handicap Inventory for Adults and with its social domain.

**Conclusion:**

The Bureau Internacional d’Audiophonologie classification seems to be more appropriate than Lloyd's and Kaplan's for use in this population; its average showed correlations with hearing loss in patients with hemodialysis time ≥ 2 years and it exhibited moderate levels of correlation with the total score of Hearing Handicap Inventory for Adults and its social domain (*r* = 0.557 and *r* = 0.512).

## Resumo

**Introdução:**

A associação entre perda auditiva e doença renal crônica e hemodiálise tem sido bem documentada. Porém, a classificação utilizada para o grau da perda pode subestimar o real diagnóstico devido a características específicas em relação às frequências auditivas mais acometidas. Além disso, correlações da perda auditiva e do tempo de hemodiálise com o handicap auditivo permanecem desconhecidas nessa população.

**Objetivo:**

Comparar os resultados das classificações de Lloyd e Kaplan e do *Bureau Internacional d’Audiophonologie* em pacientes com doença renal crônica e correlacionar as médias calculadas por suas fórmulas com o tempo de hemodiálise e com o handicap auditivo.

**Método:**

Estudo analítico, observacional e transversal com 80 pacientes em hemodiálise. Todos os pacientes foram submetidos a timpanometria, logoaudiometria, audiometria tonal limiar, e os pacientes com perda auditiva foram entrevistados através do *Hearing Handicap Inventory for Adults*. A classificação dos casos foi feita de acordo com o grau da perda. Foram verificadas as correlações das médias tonais com o tempo de hemodiálise e com as pontuações totais do *Hearing Handicap Inventory for Adults* e seus domínios.

**Resultados:**

Em 48 pacientes que responderam ao *Hearing Handicap Inventory for Adults*, 86 orelhas (53,75%) apresentaram perda auditiva em pelo menos uma das médias tonais. A Classificação do *Bureau Internacional d’Audiophonologie* identificou maior número de casos (n = 52) que apresentavam algum grau de deficiência do que a classificação de Lloyd e Kaplan (n = 16). No grupo com tempo de hemodiálise a partir de dois anos, houve correlação fraca, mas estatisticamente significante, da média da classificação do *Bureau Internacional d’Audiophonologie* com o tempo de hemodiálise (r = 0,363). Houve correlações moderadas das médias da classificação do *Bureau Internacional d’Audiophonologie* (r = 0,510) e tritonal 2 (r = 0,470) com pontuações totais do *Hearing Handicap Inventory for Adults* e com seu domínio social.

**Conclusão:**

A classificação do *Bureau Internacional d’Audiophonologie* mostra-se mais adequada do que a de Lloyd e Kaplan nessa população, sua média apresentou correlações com perdas auditivas em pacientes com tempo de hemodiálise ≥ 2 anos e manteve níveis moderados de correlação com a pontuação total do *Hearing Handicap Inventory for Adults* e seu domínio social (r = 0,557 e r = 0,512).

## Introduction

Currently, there are several classification scales of the degree of hearing loss and their formulas consider different hearing frequencies to calculate the tone average. There is not yet consensus on which scale better fits the pattern of hearing loss occurring in patients with chronic kidney disease (CKD) and hemodialysis (HD).

The best-known association between CKD and hearing loss is Alport Syndrome which has a genetic cause.^1^ However, most hearing losses that occur in CKD are not genetic, and are due to anatomical, physiological, pathological and pharmacological similarities between the nephron and vascular stria of the cochlea.^2^ The prevalence of hearing impairment is greater in CKD than in the general population,^3^ even in children,^4^, ^5^, ^6^, ^7^ and is the most severe and sensorineural in type at high frequencies.^8^, ^9^, ^10^

The worldwide prevalence of CKD has increased in recent decades. In 2013, 2.5 million patients were on dialysis in the world, and this number is expected to reach 6.5 million in 2030.^11^ In 2014, the estimated total number of dialysis patients in Brazil was 112,004, with 91.4% being on HD, and 8.6% on peritoneal dialysis.^12^

Hearing loss can affect quality of life and limits activity or restricts participation in daily activities; according to the World Health Organization (WHO), “hearing handicap” (restriction of participation) refers to the involvement in life situations and shows the individual's adaptation to the environment as a result of hearing loss and disability.^13^

Emotional and social damage from hearing impairment are variable and depend on life experiences, expectations related to health, and even on the adaptive capacity of the individual. Thus, people with similar hearing loss can experience different communicative, social and emotional difficulties in daily life and have different perceptions of their quality of life.^14^

HHIA Questionnaire (Hearing Handicap Inventory for Adults) is a tool to assess the impact of hearing loss based on the perception of hearing handicap, and among its uses in clinical practice is the ability to assess the impact of a therapeutic measure (e.g. hearing aids) and to identify specific treatment needs.^15^

As the hearing loss associated with CKD is more pronounced at high frequencies, the formula used to calculate the tone average can lead to different categorizations. Because of the importance of high frequencies in speech intelligibility, it is necessary to better understand the formula that possibly has associations with time of hemodialysis and with hearing handicap. There were no studies in the literature evaluating hearing handicap in HD patients. Thus, the objectives of this study are: (1) to compare the results of Lloyd and Kaplan and BIAP classifications, (2) to correlate the tonal averages calculated by the formulas used by these two classifications and tritonal formula of high frequencies with hemodialysis time (HT) in groups of patients with either less than 2 years and with at least 2 years of treatment, and finally (3) to correlate these averages with hearing handicap.

## Methods

This is an analytical, observational and cross-sectional study. The sample consisted of 80 patients on HD for at least three months, with ages between 14 and 54 years.

The protocol of this research is based on Resolution No. 466/12 of the National Health Council of the Ministry of Health for research with human subjects, and was approved by the Research Ethics Committee with No. 1290310/2015.

The patients selected were those on regular hemodialysis treatment in the center of nephrology of a hospital with public and private care. Inclusion criteria were: patients with CKD under the age of 55 and on HD for at least 3 months, while the exclusion criteria were: hearing loss of any etiology beginning before the CKD, transplantation, chronic ear infection, exposure to noise, mental disability, and use of ototoxic drugs for more than 1 week. Patients with normal otoscopy underwent tympanometry with an Interacoustics^®^ brand immittanciometer, model at235 XP, serial number 206331, contralateral TDH39 handset, ipsilateral clinical headset; patients with tympanogram of Jerger type “A” (1970) underwent speech audiometry and Pure Tone Audiometry (PTA) in an audiometric 2 m × 2 m cabin to assess the frequencies of 0.25; 0.5; 1; 2; 4; 6 and 8 kHz with Interacoustics^®^ audiometer, ac33 model, serial number 185994 with bone vibrator b-71, TDH-39 right and left headphones properly calibrated according to standards ISO 389-1 and ISO 389-3; PTA tests, speech audiometry and imitanciometry tests were performed by the same physician in all cases. The following average values were calculated with the formulas: tritone 1 average – Lloyd and Kaplan (0.5, 1 and 2 kHz), quadritonal average – BIAP (0.5, 1, 2 and 4 kHz) and tritonal 2 average (4, 6 and 8 kHz). Hearing losses were considered when the threshold of tritonal averages were above 25 dB HL, and BIAP average was above 20 dB HL according to the Lloyd and Kaplan (1978) and BIAP (1997) classifications, respectively. Patients with losses of these averages were interviewed for completing the HHIA that consisted of 25 questions quantifying social (12 questions) and emotional (13 questions) effects arising from hearing loss in individuals younger than 65 years. The respondent is asked to answer “yes”, “sometimes” or “no.” A “yes” answer is worth 4 points, “sometimes” is worth 2 points, and “no” does not get any points. The score ranges from 0 to 100. The classification follows the total score of HHIA: no perception of hearing handicap (0–16%), mild/moderate perception (18–42%) and severe (above 42%) perception.

Two groups of patients were created according to HT: Group I (less than 2 years) and Group II (2 years or longer).

The correlations of the three tonal averages with hemodialysis time in Groups I and II were checked. Correlations of the three tonal averages with the total scores of HHIA and their domains were also tested.

Statistical analysis was conducted using the Statistical Package for the Social Sciences – SPSS version 21.0 for Windows^®^. The profile of hearing loss and the perception of hearing handicap were established by applying descriptive statistics techniques, and the results were expressed in the form of tables and illustrative chart. The normality test used was the Shapiro–Wilk. Correlations were performed using bivariate correlation test with degree of linear relationship analyzed by Spearman coefficient. Differences were considered significant for the error value of *α* = 0.05.

## Results

**Figure 1:**
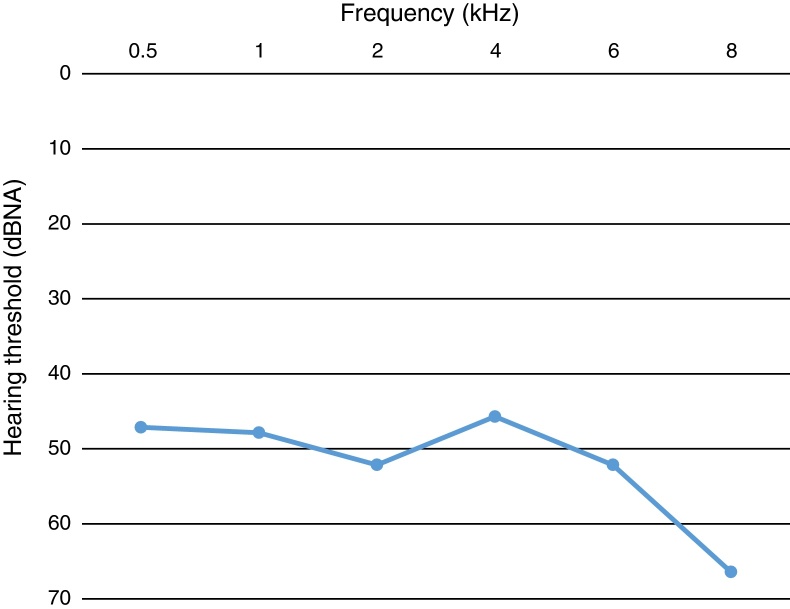
Profile of hearing loss according to average hearing threshold in frequencies.

The sample consisted of 80 patients (160 ears) on HD, 39 men (48.75%) and 41 women (51.25%). Eighty-six ears (53.75%) had hearing loss in at least one of the pure tone averages in 48 patients (uni- and bilateral loss). The 48 patients responded to HHIA, 28 (58.3%) being male. HD average time was 50.50 (± 41.25) months of the sample and of the patients with hearing loss was 54.25 (± 52) months. The type of loss was sensorineural in all cases, and with greater impairment of high frequencies (Fig. 1).

The total number of ears with losses and the distribution of grades according to Lloyd and Kaplan, BIAP and tritone 2 sensorineural can be seen in Table 1.

**Table 1:** Total of ears with hearing loss and the degree according to Lloyd's and Kaplan's and BIAP's classifications.

Tonal averages	Ears with hearing loss	Mild degree	Moderate degree	Severe degree	Profound degree	Cofosis
Tritonal 1 (0.5; 1 and 2 kHz)	16	13	2	0	1	0
BIAP average (0.5; 1; 2 and 4 kHz)	52	47	4	0	1	0
Tritonal 2 (4.6 and 8 kHz)	57	25	26	5	1	0

The correlation test between tritonal and BIAP averages with HT in Groups I and II was performed. In Group I, no correlation of averages with HT was observed. In Group II, there was a weak, but statistically significant correlation of BIAP average with HT (Table 2).

**Table 2:** Correlations between the tonal averages and the time of hemodialysis of Groups I and II.

Averages	Group I	Group II
	TH	TH
Tritonal 1 average (0.5; 1 and 2 kHz)	*p* = 0.511; *r* = 0.156	*p* = 0.335; *r* = 0.163
BIAP average (0.5; 1; 2 and 4 kHz)	*p* = 0.801; *r* = 0.060	*p* = 0.027; *r* = 0.363
Tritonal 2 average (4; 6 and 8 kHz)	*p* = 0.644; *r* = 0.110	*p* = 0.134; *r* = 0.251

Forty-eight HHIA questionnaires were filled and 43.75% had some degree of handicap perception with a total average of 30.50–63.00 (Table 3).

**Table 3:** Number of cases, percentages, total averages and of emotional and social domains according to the degree of perception of hearing handicap measured by HHIA.

Perception	*n*	%	HHIA average	HHIA (e) average	HHIA (s) average
No perception	27	56.25	6.00 (±7.65)	3.5 (±4.12)	9.00 (±8.24)
Mild/moderate perception	17	35.41	30.50 (±11.12)	15.50 (±9.00)	15.00 (±2.58)
Severe perception	04	8.34	63.00 (±7.57)	30.50 (±2.51)	32.50 (±5.98)
Total	48	100			

HHIA, *Hearing Handicap Inventory for Adults*; HHIA (e), emotional domain; HHIA (s), social domain.

There were moderate correlations, and of tritone 1 average, with the score of the social domain of HHIA, and moderate correlation of quadritonal and tritonal 2 averages with total scores of HHIA and its social domain (Table 4).

**Table 4:** Correlations between tonal averages (dBNA) with total scores of HHIA and its domains.

Averages	HHIA	HHIA (e)	HHIA (s)
Tritonal 1 averages (0.5; 1 and 2 kHz)	*p* = 0.108; *r* = 0.344	*p* = 0.468; *r* = 0.159	*p* = 0.025; *r* = 0.466
BIAP averages (0.5; 1; 2 and 4 kHz)	*p* = 0.013; *r* = 0.510	*p* = 0.066; *r* = 0.389	*p* = 0.006; *r* = 0.557
Tritonal 2 averages (4; 6 and 8 kHz)	*p* = 0.024; *r* = 0.470	*p* = 0.072; *r* = 0.382	*p* = 0.013; *r* = 0.512

HHIA, *Hearing Handicap Inventory for Adults*; HHIA (e), emotional domain; HHIA (s), social domain.

## Discussion

There was a slight predominance of females in the sample (51.25%), but men (58.3%) predominated among patients with hearing loss. The average time of HD of patients with loss (54.25 months) was higher than the average sample time (50.50 months).

When using BIAP and Lloyd and Kaplan classifications, significant differences were observed in the number of cases classified as to the degree of loss for each of them. BIAP classification identified a larger number of cases (*n* = 52) with some degree of disability compared to the classification of Lloyd and Kaplan (*n* = 16) that underestimated the number of cases. It was noted that this figure was even higher if Lloyd and Kaplan parameters were applied for the upper tritonal average (4, 6 and 8 kHz). Hearing loss in CKD has the same characteristic of loss in the elderly when a higher incidence of high frequencies occurs; BIAP classification proved to be the one that best represents the degree of hearing loss in this population with losses with this feature.^16^

Since the most pronounced hearing loss in high frequencies stands as a characteristic of loss in this population, the formula used by BIAP classification was more appropriate than the Lloyd Kaplan because it considered the high frequencies in its calculation.

In Group I no correlation of any of the tonal averages with HT was observed; however, in Group II (HT ≥ 2 years) the losses in BIAP average showed correlations with the HT. It follows that after 2 years of treatment, there was a greater impact of factors related to CKD/hemodialysis on hearing function, and this is consistent with other studies that have shown that hearing loss may occur more frequently after 2 years of HD.^17^, ^18^

Hearing handicap proved to be mild/moderate in most cases with great similarity of scores in their fields.

No level of correlation of tritonal 1 average with hearing handicap was observed. Lima et al.^19^ tested the correlations of tritonal 1 average with total HHIA and found weak but significant correlations (*r* = 0.30); these data suggest that the classification of Lloyd and Kaplan is limited because it does not reflect the impairment in communicative performance generated by such losses in this population. Total HHIA showed moderate correlations with the averages that consider the high frequencies (BIAP and tritonal 2) and the social domain portion of the HHIA. This illustrates the importance of high frequencies in the intelligibility of words, especially for the social life. Kielinen and Nerbonne^20^ identified poor correlations of the audiometric averages with hearing handicap (*r* = 0.41), but the correlations improved when the averages included 4 kHz in the formula (*r* = 0.55). Stewart et al.^21^ found a higher correlation of audiometric average of frequencies of 1–4 kHz with hearing handicap (*r* = 0.678) than the average of the frequencies of 0.5, 1 and 2 kHz (*r* = 0.550); the same authors state that professionals who use tonal averages calculated from higher frequencies tend to intervene earlier in populations with hearing loss at high frequencies through the use of sound amplification or other auditory rehabilitation procedures such as those using formulas employing 0.5 kHz.

## Conclusion

In patients with hearing loss associated with CKD/hemodialysis the BIAP classification seems to be more suitable since it allows a better categorization regarding the degree of impairment. Its calculated average through the quadritonal formula correlated with the duration of hemodialysis in patients with 2 or more years of treatment, and had moderate correlation with the total score of HHIA and its social domain.

## Conflicts of interest

The authors declare no conflicts of interest.
